# Response of Lignin Metabolism to Light Quality in Wheat Population

**DOI:** 10.3389/fpls.2021.729647

**Published:** 2021-09-13

**Authors:** Chunhui Li, Yongli Luo, Min Jin, Shufang Sun, Zhenlin Wang, Yong Li

**Affiliations:** State Key Laboratory of Crop Biology, Agronomy College of Shandong Agricultural University, Tai’an, China

**Keywords:** wheat, light quality (red/far-red), lignin biosynthesis, lodging resistance, gene regulation, lignin subunits

## Abstract

The low red/far-red (R/FR) light proportion at the base of the high-density wheat population leads to poor stem quality and increases lodging risk. We used Shannong 23 and Shannong 16 as the test materials. By setting three-light quality treatments: normal light (CK), red light (RL), and far-red light (FRL), we irradiated the base internodes of the stem with RL and FRL for 7h. Our results showed that RL irradiation enhanced stem quality, as revealed by increased breaking strength, stem diameter, wall thickness and, dry weight per unit length, and the total amount of lignin and related gene expression increased, at the same time. The composition of lignin subunits was related to the lodging resistance of wheat. The proportion of S+G subunits and H subunits played a key role in wheat lodging resistance. RL could increase the content of S subunits and G subunits and the proportion of S+G subunits, reduce the proportion of H subunits. We described here, to the best of our knowledge, the systematic study of the mechanism involved in the regulation of stem breaking strength by light quality, particularly the effect of light quality on lignin biosynthesis and its relationship with lodging resistance in wheat.

## Introduction

Wheat (*Triticum aestivum* L.) is one of the most important crop in the world, and it plays a crucial role in determining global food security (Zhang et al., 2020). As the global population grows, the production of wheat needs to increase by 70% to ensure food security by 2050 (Curtis and Halford, 2014). Wheat yield needs to be achieved by increasing planting density and fertilization (Xiao et al., 2015; Fang et al., 2018). However, these factors increase competition for water, fertilizer, and light within the population, thus increasing the risk of lodging. Lodging affects not only crop yield but also crop quality and increases the cost of harvesting. Therefore, how to improve the stem quality, especially the breaking strength of stem base internodes, has become the main goal to improve the lodging resistance and yield of wheat.

The basal internodes of the wheat plant, especially the second internode (counted up from the surface), have the greatest influence on the lodging resistance (Zhang et al., 2017). The breaking strength of the basal internodes significantly influences the performance of wheat in terms of lodging resistance, which is significantly correlated with stem diameter, wall thickness and, dry weight per length (Meng et al., 2016; Zhang et al., 2016). Lignin is a structural component of the secondary cell wall of plants, which plays an important role in plant growth, lodging resistance, and various stresses (Hussain et al., 2019). It is closely related to the breaking strength of the stem (Liu et al., 2019). Lignin is composed of three structural subunits, sinapyl alcohol, p-hydroxyphenyl alcohol, and coniferyl alcohol, which form syringyl (S), p-hydroxyphenyl (H), and guaiacyl (G) through polymerization reactions (Li et al., 2008; Nguyen et al., 2016). The lignin subunits structure is highly conserved, but its content and proportion are strongly influenced by the environment (Hu et al., 2016).

Light plays a wide range of roles in regulating plant growth, morphogenesis, and physiological metabolism. Photomodulation is an economical and environmentally friendly method to provide a suitable light environment for crops, while reducing chemical fertilizer inputs, improving quality and efficiency to ensure ecological safety (Huang et al., 2015; Li et al., 2018). Evidence is growing that shade avoidance responses (reduced stem elongation and diameter) occur in crops when the lower plant canopy has a low red/far-red (R/FR) light ratio (Child et al., 2010; Jin et al., 2021). Plant cells regulate plant growth and developmental processes mainly through the effects of light interactions, such as those mediated by phytochrome signaling pathways. Regulation of intermediate or end products of the photosensitized pigment signaling pathway significantly affects plant growth (Cao et al., 2016; Legris et al., 2019). Plant height is largely influenced by endogenous hormones, among which, gibberellin (GAs) plays an important role in stem elongation (Huang et al., 2019; Tang et al., 2020). In both rice (*Oryza sativa Linn*.) and *Arabidopsis* [*Arabidopsis thaliana (Linn.) Heynh*.], GAs synthesis gene mutants show dwarfism, while light and GAs are antagonistic to cell elongation. Light induces the onset of photomorphogenesis and inhibited hypocotyl growth in plants, while GAs promoted yellowing growth and increased hypocotyl elongation (Lucas et al., 2008; Buss et al., 2020). Phytochrome can be divided into red-absorbing forms (Pr) and far-red absorbing forms (Pfr) according to the type of chromophore (Chen and Chory, 2011). Upon exposure to red light (RL), Pr is converted to Pfr, which is translocated to the nucleus. In the nucleus, Pfr interacts directly with the basic helix-loop-helix (bHLH) subfamily of transcription factors, called phytochrome-interacting factors (PIFs; Nito et al., 2013). PIF3, in *Arabidopsis*, which could inhibit cotyledon elongation under far-red light (FRL), but promote embryonic axis elongation under RL (Yue et al., 2015; Hernando et al., 2021). R/FR significantly affected basal internode length in rice (Zhong et al., 2020). Previous studies demonstrated that increasing the proportion of R/FR from 1.0 to 3.8 reduced the height of petunia [*Pharbitis nil (L.) Ching*] plants by 30% (Fletcher et al., 2015). In soybean [*Glycine max (Linn.) Merr*.], high density significantly reduced the proportion of R/FR at the base of the canopy, which inhibited lateral growth of soybean stems and promoted elongation of internodes at the base of the stem resulting in reduced stem thickness and increased plant height (Cheng et al., 2018; Liu et al., 2018b). Previous studies showed that low R/FR proportion conditions had important effects on lignin biosynthesis and metabolism by affecting the activities of phenylalanine ammonia-lyase (PAL), peroxidase (POD), 4-coumarate: CoA ligase (4CL), and cinnamyl alcohol dehydrogenase (CAD; Koyama et al., 2012; Liu et al., 2018a). As expected, downregulation of these enzyme genes could lead to a decrease in total lignin and alter monomeric proportions, damaged xylem and vascular bundles, and reduced stem quality and lodging resistance (Zheng et al., 2017a; Hussain et al., 2021). In addition, downregulation of ferulic acid-5-hydroxylase (F5H) and caffeic acid-oxy-methyltransferase (COMT) genes had little effect on total lignin (Lin et al., 2015), but could alter the macromolecular structure and subunit polymerization of lignin, affecting the lignin subunit proportion and thus the breaking strength of the stem (Baucher et al., 2003; Stewart et al., 2009). The relationship between wheat varieties’ lodging resistance and stem morphological and mechanical traits had been investigated more frequently, but very little was found in the literature on the question of the effect of basal R/FR proportion on lodging resistance and lignin subunits content and proportion in wheat. By setting up different light quality treatments, we investigated the regulatory mechanism of different light quality on wheat stem traits and lignin synthesis, aiming to provide a theoretical basis and technical support for enhancing wheat’s resistance to lodging.

## Materials and Methods

### Experimental Design

The experiment was conducted at the experimental station of Shandong Agricultural University, Tai’an, China (36° 9' N, 117° 9' E; altitude 128m) during the 2018–2019 and 2019–2020 growing seasons. The experimental land was sandy loam with organic matter of 15.8g kg^−1^, total nitrogen of 1.34g kg^−1^, nitrate nitrogen of 14.21mg kg^−1^, ammonium nitrogen of 6.58mg kg^−1^, available phosphorus of 8.87mg kg^−1^, and available potassium of 84.62mg kg^−1^ in the 0–20cm soil layer.

**Figure 1:**
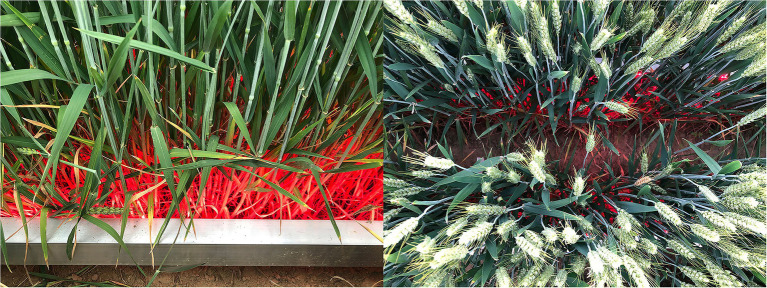
Supplementary light irradiation device.

Two typical cultivars of winter wheat currently used in local production, Shannong16 (SN16) and Shannong23 (SN23), were utilized in this study. SN16 is a lodging-sensitive cultivar, and SN23 is a lodging-resistant cultivar. Three light quality treatments, normal light (CK), RL and, FRL, were set up for the experiment, and the basal internodes of stems were given RL (660nm, 1.3>R/FR>1.6) for 7h and far-red light (720nm, 0.3>R/FR>1.0) for 7h daily from 9:00 to 16:00 from the beginning of jointing (GS 31; Zadoks et al., 1974) to flowering (GS 65), respectively. The light device was installed at 5cm from the ground surface with the light source perpendicular to the stalk, as shown in Figure 1. The plot size was 9m^2^ with three replications. The treatments were planted at a density of 2.25 million plants ha^−1^. In addition, during the growing seasons, 120kg ha^−1^ N, 75kg ha^−1^ P_2_O_5_, and 150kg ha^−1^ K_2_O were applied as the basal fertilizer before planting, and 120kg ha^−1^ N was top-dressed at the jointing stage (GS 31). Pests, diseases, and weeds were well controlled to avoid yield losses.

### Breaking Strength

The breaking strength of wheat was measured at flowering (GS 65), milking (GS 75), and dough (GS 85) stages using a digital stem strength meter (Hangzhou Tepe Instrument Co., Ltd., Hangzhou, China; Hussain et al., 2020). The internodes without stem sheath were placed 5cm away from the groove of the support post. The center of the internode was aligned with the pressure probe. The breaking strength was the peak value when the internode was broken. It was displayed on the instrument screen and its unit was Newton (N).

### Measurement of Morphological Characteristics in the Second Internode of Stem

Fifteen representative main stems (removing stem sheath) were taken at flowering (GS 65), milking (GS 75), and dough (GS 85) stages, and the diameter, wall thickness, and dry weight per unit length of the second internode at the base of the stem were prepared by adapting the procedure used by Wei et al. (2008). The diameter and wall thickness of the stem were measured with a vernier caliper with an accuracy of 0.001mm; dry weight per unit length was scored with the formula (the dry weight of basal second internode/the length of basal second internode).

### Gibberellin Determination

The gibberellin content in the second internode of wheat stems was measured from the beginning of the formation of the second internode to day 35. Gibberellin acid4 (GA4) and gibberellin acid7 (GA7) were extracted according to the method described by Dave et al. (2011), with the extraction buffer (isopropanol: chromatographic acetic acid, 99:1) being used as the extractant. The qualitative and quantitative analyses of the GA4 and GA7 in the wheat stem were performed by ultra-performance liquid chromatography-electrospray tandem mass spectrometry (UPLC-MS/MS) system (ACQUITY UPLC I-Class/Xevo TQ-S, Waters Co., United States).

### Lignin Determination

The lignin content of the second internode of wheat stems was determined from the beginning of the formation of the second internode to day 49. Lignin determination was done as previously reported by Peng et al. (2014). Around 0.3g of fresh stem samples with leaf sheaths removed were ground in liquid nitrogen, rinsed five times with 80% ethanol to remove soluble metabolites, rinsed with acetone, and dried in a drying oven. Samples were exposed to a 4:1 (v/v) mixture of acetic acid: acetyl bromide and incubated at 70°C for 2h. Then, the samples were cooled to room temperature and transferred to 50ml volumetric flasks to which 2M NaOH, acetic acid, and 7.5M hydroxylamine hydrochloride were added to terminate the reaction. The fixation of each sample was completed with acetic acid. The absorbance of the samples was read at 280nm using a spectrophotometer (Beijing Pu-analysis General Instrument Co., Ltd.). Lignin content was expressed as OD280 ml^−1^ g^−1^ fresh weight (FW).

### Determination of Lignin Subunits

At flowering (GS 65), milking (GS 75), and dough (GS 85) stages, the content of the lignin subunits (S, G, and H) in the second internode of the wheat stem was determined. The content of lignin subunits was prepared according to the procedure used by Zheng et al. (2017b) with slight modifications. Lignin in wheat stems was oxidatively depolymerized to lignin subunits by alkaline nitrobenzene oxidation. The qualitative and quantitative analysis of the three lignin subunits in wheat straw was performed by UPLC-MS/MS system (ACQUITY UPLC I-Class/Xevo TQ-S, Waters Co., United States). A dry sample of 20mg of the protein-free stem was accurately weighed in a 50ml white digestion tube; then 3ml of 2M NaOH and 0.5ml of nitrobenzene were added, and the sample was thoroughly mixed and heated in a Master 40 microwave digestion instrument (Shanghai, Xin Yi Microwave Chemical Technology Co., Ltd.) at 170°C for 1h. The reaction mixture was then centrifuged and extracted with 4ml of ethyl acetate. The supernatant was extracted twice. Ethyl acetate was evaporated using a Concentrator plus vacuum centrifuge concentrator (Eppendorf, Germany). The sample was re-dissolved with 6ml of 50% acetonitrile in water, filtered through 0.22μm membrane, and injected into 4μl for detection on the machine.

### Real-Time Quantitative PCR Assay

We searched for genes in the NCBI GenBank nucleotide database^1^ and the Ensembl plant’s database,^2^ which gave homologous gene information for a given gene, then designed primers based on the comparison results of the wheat genome sequence published by IWGSC.^3^ The primers were designed in the conserved regions of genes. Instead of amplifying a specific gene, we amplified that gene and all its homologs at the same time as a way to test the expression of all of them, since their expression products were functionally identical. Gene-specific primers with Tm ranging from 50 to 60°C and amplifying PCR products ranging from 75 to 200bp in length were designed using Primer 5.0 (Table 1). All the designed primers were tested for full efficiency before use for expression profiling of target genes. The gene expression encompassed three independent biological replicates of each treatment. For each biological replicate, three technical replicates of each PCR reaction were performed. Briefly, total RNA from the second basal internode was isolated using a modified Trizol extraction method and treated with DNase I to remove any contaminant genomic DNA. First-strand cDNA was synthesized from 1μg of total RNA using the Prime Script RT Reagent Kit (QUANSHIJIN, China).

**Table 1:** Sequence of primers and probes for *TaPhy A*, *TaPhy B*, and key genes in the lignin biosynthesis pathway.

Gene ID	Primer sequence-F (5'–3')	Primer sequence-R (5'–3')
*β-ACTIN* (TraesCS1A02G274400)	GGGACCTCACGGATAATCTAATG	CGTAAGCGAGCTTCTCCTTTAT
*Phytochrome A (PHY A1)* (TraesCS4A02G262900)	TTGTGAATGCTTGTGCCAGC	TGAGGGAGACTACATGGCGA
*Phytochrome B (PHY B3)* (TraesCS4A02G122500)	CACCAGAATCACACGCAGTC	GATCTGCTGCTCGGAGGAG
*Phenylalanine ammonialyase (PAL4)* (TraesCS6A02G222800)	CATCTTGGAGGGAAGCTCATAC	GACTTGGTGGCAAATCGAATAAC
*Cinnamate 4-hydroxylase (C4H2)* (TraesCS3B02G375100)	GCCGAGAGCAAGATCCTCGT	CGTGCTTCTCCTCCTCCAGG
*p-Hydroxycinnamoyl-CoA shikimate (HCT2)* (TraesCS2B02G374600)	TGTCAGCATTGCTCCGTGGA	CCAGGAGCCATGAACACCGG
*p-Coumarate 3-hydroxylase (C3H1)* (TraesCS3D02G336900)	CCCATCCTCGGCATCACCAT	CGCCCTTCTCAGTCGTGTCA
*Cinnamoyl-CoA reductase (CCR2)* (TraesCS5D02G232400)	CGTGATGGTGCTGAAGAAAC	CGATCATCGAAGCCGATACA
*Cinnamyl alcohol dehydrogenase (CAD4)* (TraesCS6D02G162800)	GAGGTCGTCAAGATGGAC	CTAGCTCTTTCTCCCTCTG
4-*Coumarate:CoA ligase (4CL2)* (TraesCS7D02G483400)	TGCACACTGGAGACATTGGC	TTCGAGTTCCGCAGGAGGTA
*Ferulate 5-hydroxylase (F5H2)* (TraesCS2B02G518400)	AGCTCCCCTCTCTCAAGTGC	GACACAGTCCTCGGCGTTCT
*Caffeic acid O-methyltransferase (COMT1)* (TraesCS3B02G612000)	GATCCATGACAACGAGTCTACC	CGAATCAATCGACGACACAAAC
*Caffeoyl shikimate esterase (CSE)* (TraesCS2B02G229300)	GCCAGCAAGGACAAGACCATCA	GCTGAGCCAGGCGAGGATGT

The quantitative PCR (qPCR) assay was performed on the 7,500 Real-Time PCR instrument (ABI, United Kingdom). The reaction consists of 2μl of the diluted cDNA as a template, 10μl of Top/Tip Green qPCR Supermix (QUANSHIJIN, China), 0.4μl of 10μM forward primer (200nM final concentration), 0.4μl of 10μM reverse primer (200nM final concentration), 0.4μl of passive reference dye (50×; QUANSHIJIN, China), and 6.8μl nuclease-free water, total 20μl. The samples were subjected to the following thermal cycling conditions: DNA polymerase activation at 94°C for 30s followed by 45cycles of denaturation at 95°C for 5s, annealing at 50–60°C (depending on the melting temperature of the primer set) for 15s and extension at 72°C for 10s in duplicate in 96-well optical reaction plates (ABI, United Kingdom). Relative expression was calculated using equation 2^-∆∆Ct^ (Livak and Schmittgen, 2001).

### Statistical Analysis

Tables and figures were processed with Microsoft Excel 2007 and SigmaPlot 14.0 Software. Data management and analysis were performed using Data Processing System software 7.05 (DPS) and IBM SPSS statistics 22 statistical software. The means and significant differences between treatments were separated using the least significant difference (LSD) test at 5% probability.

## Results

### The ANOVA for the Effects of Cultivar and Light Quality on Breaking Strength, the Content of S, G, and H Subunits, and Lignin the Content of Stems as Well as Dry Weight per Length, Diameter, and Wall Thickness, in Two Wheat Cultivars

The analysis of variance showed that wheat variety and light quality significantly (*p*<0.01) affected wheat stem breaking strength, dry weight per length, stem diameter, wall thickness, and S, G, and H subunit contents as well as total lignin content (Table 2). What stands out in Table 2 is the effect of variety×light quality (C×L) on breaking strength, S, G, and H subunit contents were significant (*p*<0.01). However, the effect of variety×light quality (C×L) on dry weight per length, stem diameter, wall thickness was not significant. In addition, variety×light quality (C×L) had a significant effect on total lignin content (*p*<0.05).

**Table 2:** Variance analyses for the effects of the cultivar (C) and light quality (L) on breaking strength, the content of S, G, and H subunits, and lignin content of stems as well as dry weight per length, diameter, and wall thickness, in two wheat cultivars.

Source of variation	BS	DW	D	WT	S subunits	G subunits	H subunits	Lignin
Cultivar, C	292.77^**^	377.44^**^	24.82^**^	70.12^**^	5586.15^**^	4776.07^**^	3324.14^**^	269.57^**^
Light quality, L	158.66^**^	88.49^**^	253.08^**^	81.91^**^	2166.73^**^	867.23^**^	197.62^**^	253.84^**^
C×L	26.38^**^	0.18^ns^	2.65^ns^	2.70^ns^	350.58^**^	211.79^**^	70.27^**^	3.52^*^

*F-values were shown. C, cultivar; L, quality; BS, breaking strength; DW, dry weight per length; D, diameter; WT, wall thickness; S, syringyl lignin; G, guajacyl lignin; H, p-hydroxyphenyl lignin; and Lignin, lignin content*.

** *Represent significance at the 0.01 probability level*.

* *Represent significance at the 0.05 probability level*.

ns *Represent not significant at the 0.05 probability level*.

### Response of Wheat Breaking Strength to Light Quality in Population

The results, as shown in Table 3, indicated that both varieties showed a decreasing trend in folding resistance from the flowering to the waxing stage. Further analysis showed that compared to the CK treatment, the second internode stem breaking strength was significantly increased by 54.45, 46.69, and 34.73% in the three periods of the RL treatment in 2018–2019 and by 47.44, 49.03, and 36.74% in 2019–2020 (mean of both varieties), and the three stages of the FRL treatment in 2018–2019 times had a significant reduction of 21.55, 31.51, and 43.89% in the second internode breaking strength of the stem; and 26.84, 31.24, and 44.01% in 2019–2020 (mean of both varieties).

**Table 3:** Effect of light quality in wheat population on breaking strength in 2018–2019 and 2019–2020 growing seasons.

Cultivars	Treatments	2018–2019			2019–2020		
		Anthesis (N) (DC, 65)	Milk (N) (DC, 75)	Dough (N) (DC, 85)	Anthesis (N) (DC, 65)	Milk (N) (DC, 75)	Dough (N) (DC, 85)
SN23	CK	8.08±0.72^b^	7.90±0.79^b^	6.23±0.36^b^	9.35±0.35^b^	7.93±0.30^b^	6.12±0.14^b^
	RL	14.87±0.49^a^	11.24±0.63^a^	8.50±0.56^a^	15.66±0.37^a^	11.36±0.36^a^	8.54±0.31^a^
	FRL	6.33±0.18^c^	4.83±0.58^c^	4.20±0.33^c^	6.57±0.30^c^	4.84±0.28^c^	4.17±0.37^c^
SN16	CK	6.48±0.47^b^	3.72±0.35^b^	3.42±0.36^b^	6.49±0.13^b^	3.66±0.11^b^	3.40±0.12^b^
	RL	7.63±0.54^a^	5.82±0.40^a^	4.51±0.50^a^	7.69±0.17^a^	5.92±0.13^a^	4.47±0.17^a^
	FRL	5.09±0.64^c^	3.13±0.18^c^	1.21±0.17^c^	5.02±0.20^c^	3.14±0.13^c^	1.16±0.12^c^

*SN16 and SN23 represent the lodging-sensitive cultivar Shangnong16 and the lodging-resistant cultivar Shangnong23. CK represents the normal light, RL represents the red light, and FRL represents far-red light. Data are the means±SD of 10 replicates. Values followed by different letters are significantly different at p<0.05 (n=10)*.

### Response of Wheat Stem Dry Weight per Length to Light Quality in Population

Dry weight per length in the wheat stem of both varieties showed a trend of increasing and then decreasing from anthesis to dough stage. It was apparent from Table 4 that compared to the CK treatment, the dry weight per length in wheat stem under the RL treatment increased by 13.10, 7.23, and 18.73% in 2018–2019; 26.15, 26.44, and 26.09% in 2019–2020 (mean of both varieties), respectively, and the dry weight per length in wheat stem under the FRL treatment in 2018–2019 decreased by 8.81, 3.44, and 9.92%, respectively; and 8.92, 17.07, and 10.51% (mean of both varieties) from 2019 to 2020, respectively. In addition, dry weight per length in the wheat stem was significantly higher in SN23 than in SN16 (Table 4).

**Table 4:** The effect of light quality in population on dry weight per length in wheat stem in 2018–2019 and 2019–2020 growing seasons.

Cultivars	Treatments	2018–2019			2019–2020		
		Anthesis (mgcm^−1^) (DC, 65)	Milk (mgcm^−1^) (DC, 75)	Dough (mgcm^−1^) (DC, 85)	Anthesis (mgcm^−1^) (DC, 65)	Milk (mgcm^−1^) (DC, 75)	Dough (mgcm^−1^) (DC, 85)
SN23	CK	0.0268±0.0015^b^	0.0338±0.0004^b^	0.0238±0.0004^b^	0.0212±0.0004^b^	0.0258±0.0002^b^	0.017±0.0004^b^
	RL	0.0296±0.0012^a^	0.0354±0.0010^a^	0.0261±0.0017^a^	0.0241±0.0001^a^	0.0308±0.0005^a^	0.0202±0.0003^a^
	FRL	0.0237±0.0018^c^	0.0331±0.0007^c^	0.0208±0.0015^c^	0.0195±0.0003^c^	0.0221±0.0004^c^	0.0151±0.001^c^
SN16	CK	0.0152±0.0006^b^	0.0215±0.0011^b^	0.0125±0.0004	0.0113±0.0002^b^	0.0158±0.0002^b^	0.0106±0.0002^b^
	RL	0.0179±0.0007^a^	0.0239±0.0007^a^	0.017±0.0011^a^	0.0169±0.0004^a^	0.0218±0.0003^a^	0.0146±0.0001^a^
	FRL	0.0146±0.0009^b^	0.0203±0.0003^c^	0.0119±0.0009^c^	0.0101±0.0002^c^	0.0124±0.0002^c^	0.0096±0.0004^c^

*SN16 and SN23 represent the lodging-sensitive cultivar Shangnong16 and the lodging-resistant cultivar Shangnong23. CK represents the normal light, RL represents the red light, and FRL represents the far-red light. Data are the means±SD of 10 replicates. Values followed by different letters are significantly different at p<0.05*.

### Response of Wheat Stem Diameter and Wall Thickness to Light Quality in Population

**Figure 2:**
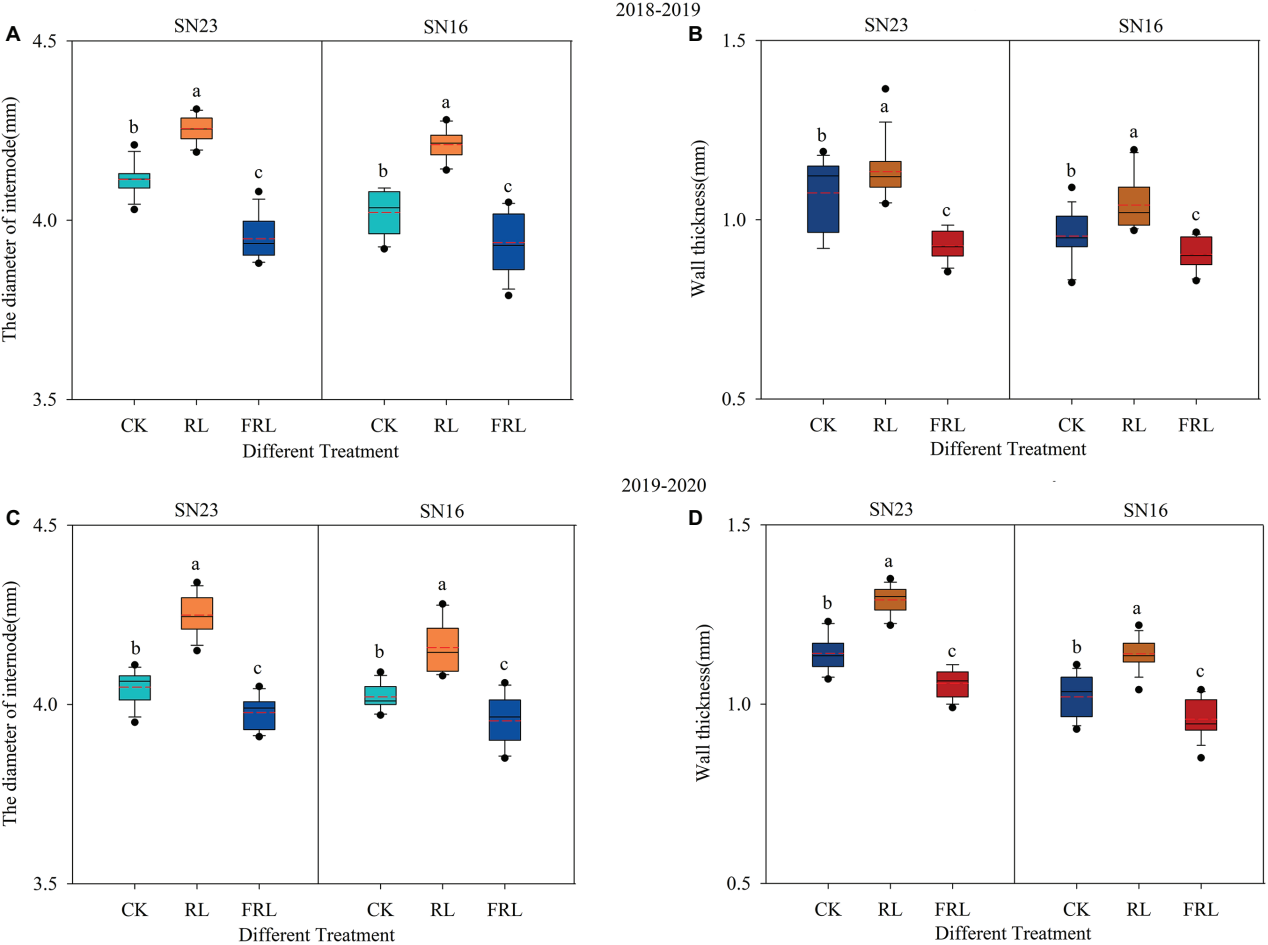
The effect of light quality in population on diameter **(A,C)** and wall thickness **(B,D)** in wheat in 2018–2019 and 2019–2020 growing seasons, respectively. SN16 and SN23 represent the lodging-sensitive cultivar Shangnong16 and the lodging-resistant cultivar Shangnong23. CK represents the normal light, RL represents the red light, and FRL represents far-red light. Solid lines and red dotted lines indicate medians and means, respectively; box boundaries indicate upper and lower quartiles, whisker caps indicate 90th and 10th percentiles (*n*=12). Significant differences between treatments according to the least significant difference (LSD; *p*<0.05).

We could see that R/FR had significant effects on the second internode diameter and wall thickness at the base of the stem, and both increased with the increase in the proportion of R/FR (Figure 2). As can be seen in Figure 2, compared to the CK treatment, the second internode diameter of the RL treatment increased by 4.06% and the FRL treatment decreased by 3.07% in 2018–2019, and in 2019–2020, the second internode diameter of the RL treatment increased by 4.19% and the FRL treatment decreased by 1.70% (mean of both varieties). Compared to the CK treatment, the wall thickness of the second internode of the plants increased by 6.44% in the RL treatment and decreased by 11.59% in the FRL treatment in 2018–2019, and increased by 12.45% in the RL treatment and decreased by 6.74% in the FRL treatment in 2019–2020 (mean of both varieties). Additionally, diameter and wall thickness were significantly higher in SN23 than in SN16.

### Response of Gibberellin (GA4, GA7) to Light Quality in Population

**Figure 3:**
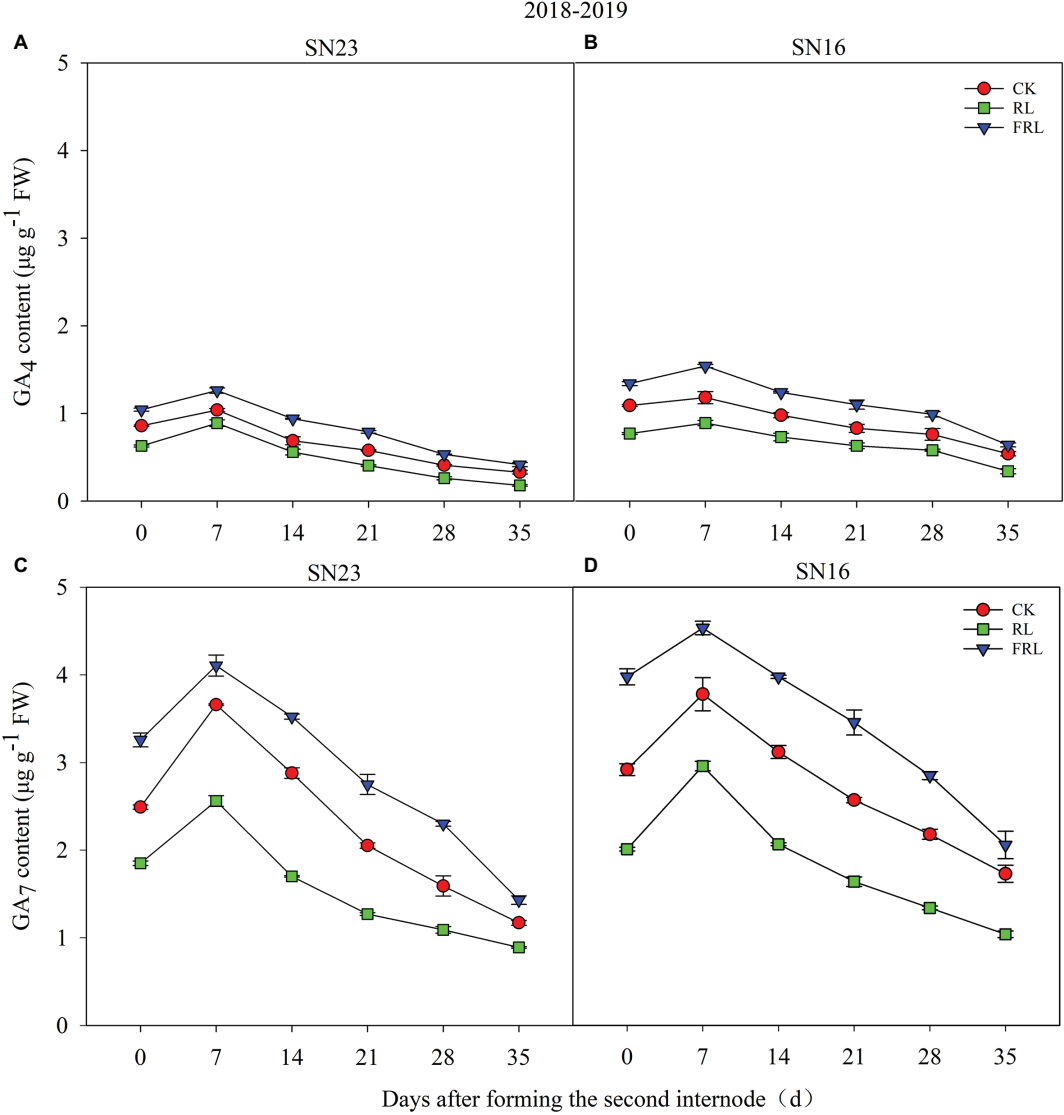
The effect of light quality in population on GA4 **(A,B)** and GA7 **(C,D)** in wheat in 2018–2019 growing seasons. SN16 and SN23 represent the lodging-sensitive cultivar Shangnong16 and the lodging-resistant cultivar Shangnong23. CK represents the normal light, RL represents the red light, and FRL represents far-red light. Vertical bars above mean values indicate SDs (*n*=3). Means show significant differences between treatments according to the LSD (*p*<0.05).

It could be seen from the data in Figure 3 that the light quality within the population significantly affected the gibberellin content (GA4, GA7) of wheat stems, with GA7 content significantly higher compared to GA4 (Figure 3). Compared with the CK treatment, the RL treatment significantly reduced the GA4 and GA7 content and the FRL treatment significantly increased the GA4 and GA7 content. Further analysis showed that GA4 and GA7 contents showed a trend of increasing and then decreasing with time and reached the maximum value 7days after the formation of the second node at the base of the wheat stem. In addition, the gibberellin content (GA4, GA7) of SN23 was always lower than that of SN16.

### Response of Lignin Accumulation in Wheat Stems to Light Quality in Population

**Figure 4:**
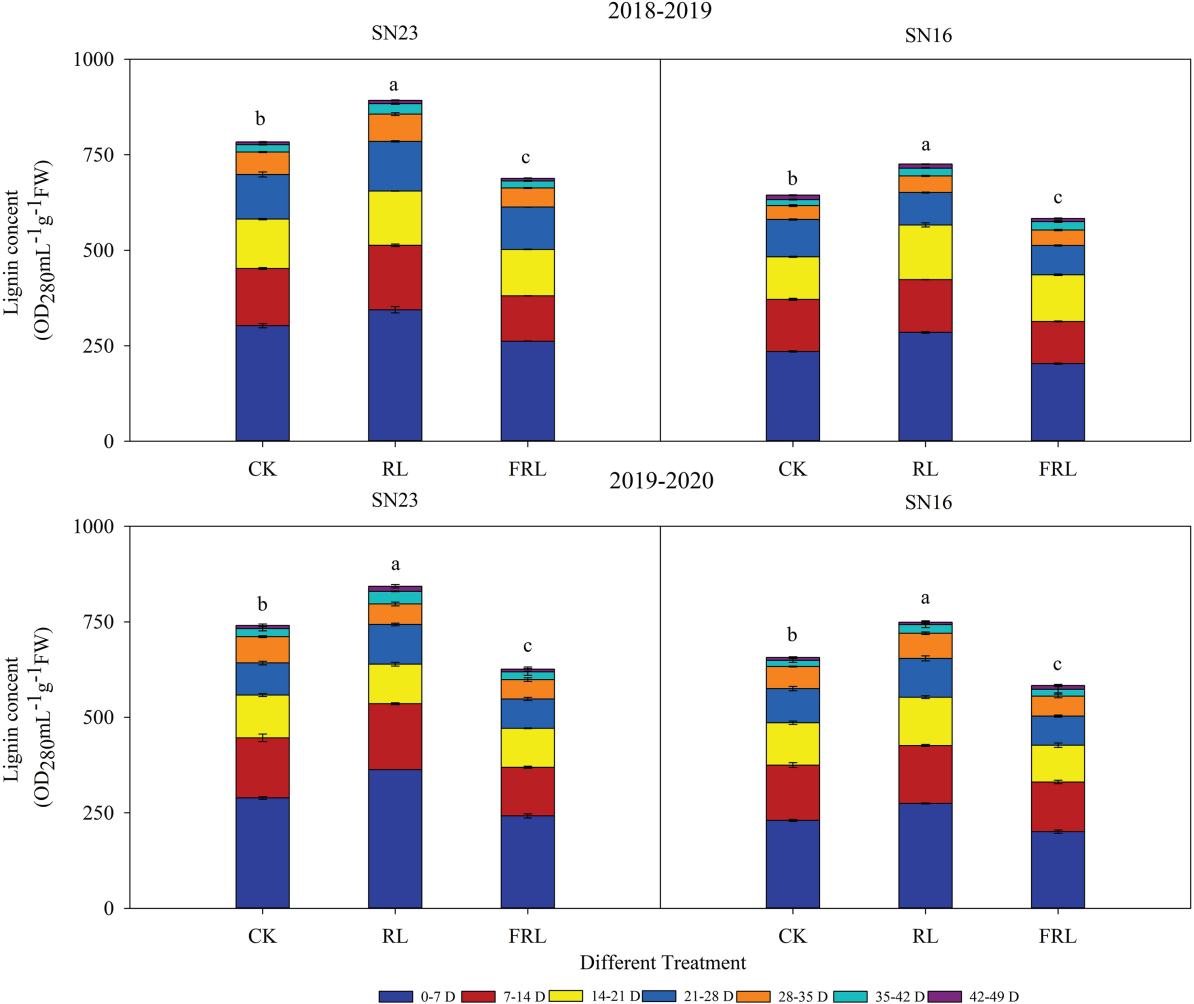
The effect of light quality in population on lignin accumulation in wheat in 2018–2019 and 2019–2020 growing seasons, respectively. SN16 and SN23 represent the lodging-sensitive cultivar Shangnong16 and the lodging-resistant cultivar Shangnong23. CK represents the normal light, RL represents the red light, and FRL represents far-red light. Different colors represent the days after the second internode formation in the 2018–2019 and 2019–2020 growing seasons. FW, fresh weight. Vertical bars above mean values indicate SDs (*n*=3). Means show significant differences between treatments according to the LSD (*p*<0.05).

Lignin accumulation during wheat stem development was determined according to the effect of different R/FR conditions on the degree of lignification of the second internode of wheat stems (Figure 4). In 2018–2019, the total lignin accumulation under the RL treatment increased by 13.34% compared with the CK treatment; the FRL treatment decreased by 10.91% compared with the CK treatment (CK; mean of both varieties); in 2019–2020, the total lignin accumulation under the RL treatment increased by 13.95%; the FRL treatment decreased 13.44% compared to the CK treatment (CK; mean of both varieties). Figure 4 showed that there were differences in lignin accumulation during stem development in winter wheat, with faster accumulation rates from 0 to 35 days after the formation of the second internode. These results suggested that the jointing and anthesis stages were critical periods for lignin accumulation. In addition, the trend of lignin accumulation was the same in both varieties, but the lignin accumulation of SN23 was higher than that of SN16, under different treatments.

### Response of Lignin Subunit Content and Proportion to Light Quality in Population

**Figure 5:**
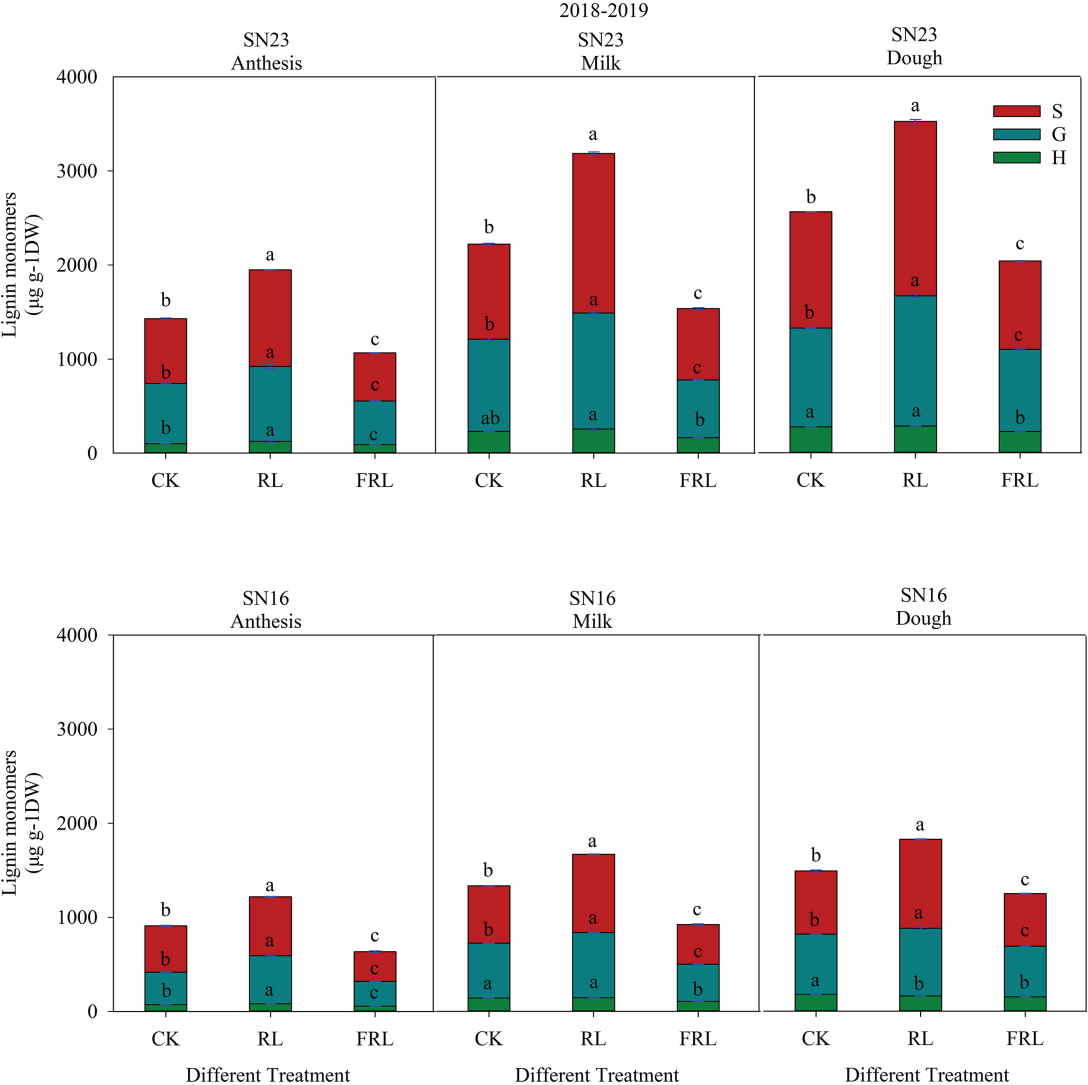
The effect of light quality in population on lignin subunit content in wheat in 2018–2019 growing seasons. SN16 and SN23 represent the lodging-sensitive cultivar Shangnong16 and the lodging-resistant cultivar Shangnong23. CK represents the normal light, RL represents the red light, and FRL represents far-red light. H, G, and S represent p-hydroxyphenyl lignin, guajacyl lignin, and syringyl lignin, respectively. Vertical bars above mean values indicate SDs (*n*=3). Means show significant differences between treatments according to the LSD (*p*<0.05).

As shown in Figure 5, wheat stem lignin consisted of S, G, and H subunits, with S and G subunits being the most abundant and H subunits accounting for the smallest percentage. The total lignin content and subunit content increased with fertility, and the total lignin content and subunit content of SN23 were higher than those of SN16, and both responded to RL and FRL with similar trends. In 2018–2019, compared with the CK treatment, the total lignin subunit content of the RL treatment significantly increased by 35.28, 36.54, and 31.95% (mean of both varieties); the FRL treatment significantly decreased by 27.38, 30.88 and 18.84% (mean of both varieties).

**Figure 6:**
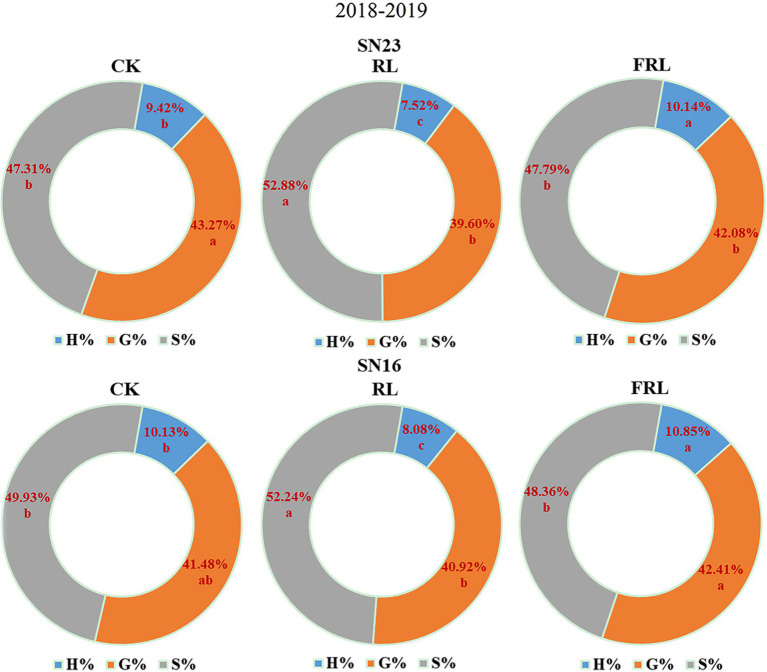
The effect of light quality in population on lignin subunit percentage at the duogh stage in wheat in 2018–2019 growing seasons. SN16 and SN23 represent the lodging-sensitive cultivar Shangnong16 and the lodging-resistant cultivar Shangnong23. CK represents the normal light, RL represents the red light, and FRL represents far-red light. H, G, and S represent p-hydroxyphenyl lignin, guajacyl lignin, and syringyl lignin, respectively.

As could be seen from Figure 6, in 2018–2019, compared with the CK treatment, the RL treatment significantly increased the content of S and G subunits and the proportion of S+G subunits, and significantly decreased the proportion of H subunits; the FRL treatment significantly decreased the content of S and G subunits and the proportion of S+G subunits, and significantly increased the proportion of H subunits. Overall, these results indicated that S+G and H subunit proportions play a key role in the performance of wheat in terms of resistance to lodging.

### Response of Phytochrome and Lignin Synthesis Key Enzyme Gene Expression to Light Quality in Population

**Figure 7:**
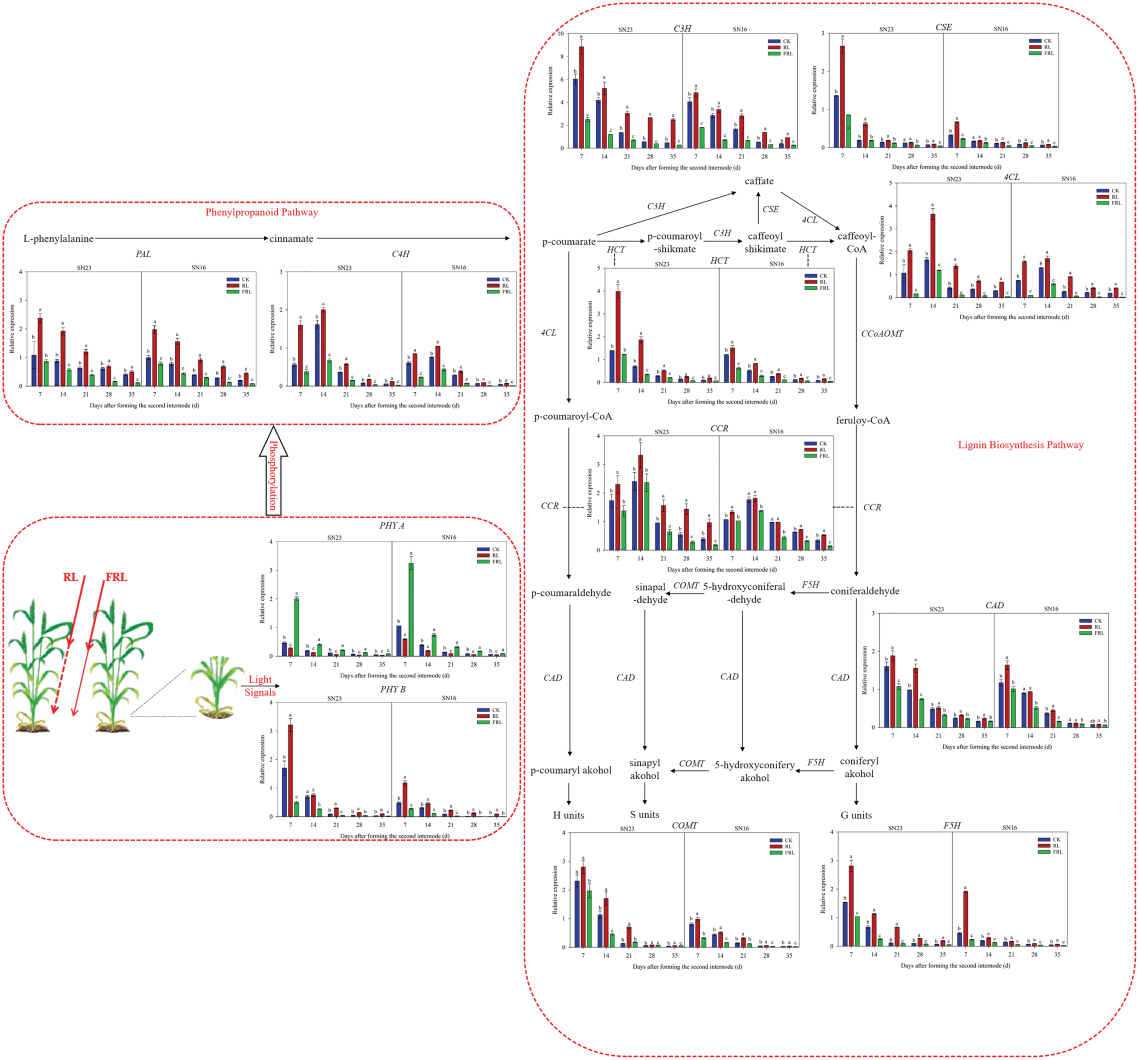
Effects of light quality on real-time quantitative PCR (RT-qPCR) of key genes for phytochrome and lignin synthesis in wheat in 2018–2019 growing seasons. SN16 and SN23 represent the lodging-sensitive cultivar Shangnong16 and the lodging-resistant cultivar Shangnong23. CK represents the normal light, RL represenst the red light, and FRL represents far-red light. Vertical bars above mean values indicate SDs (*n*=3). Means show significant differences between treatments according to the LSD (*p*<0.05).

The RL treatment significantly increased the relative expression of *TaPhy B*, which decreased gradually with the growth and development process, and the RL treatment slowed down this decrease, while the FRL treatment accelerated it (Figure 7). The RL treatment significantly decreased the relative expression of *TaPhy A*, which gradually decreased with the growth and development process, and the RL treatment accelerated this rate of decrease, while the FRL treatment slowed down this rate of decrease.

As shown in Figure 7, the transcript abundance of most of the key lignin synthesis enzymes decreased gradually with the progress of growth and development. The effect of different light qualities on the relative expression of *TaPAL* and *TaC3H* gradually increased with increasing days of light exposure, and high RL and FRL proportion conditions retarded the decreasing rate. The relative expression of *TaC4H* and *Ta4CL* genes reached the maximum at 14days after illumination, and the effect of different light quality on the relative expression of *TaC4H* and *Ta4CL* genes followed the trend: RL>CK>FRL. The relative expression of *TaHCT* and *TaCSE* genes increased significantly under high RL and FRL proportion conditions, but the differences with other treatments gradually decreased over time. *TaF5H* and *TaCOMT* were key enzymes in the lignin S subunit production step, and RL treatment significantly increased the relative expression of both genes, while the differences were not significant under the CK treatment and the FRL treatment, and with the progression of fertility. *TaCCR* and *TaCAD* are the last two steps of lignin subunit synthesis, and the relative expression of *TaCCR* gene reached the maximum after 14days of light treatment, while *TaCAD* gradually decreased with the fertility process. Compared with the CK treatment, the RL treatment significantly increased the relative expression of both genes, the FRL treatment had no significant effect on the relative expression of *TaCAD* gene, and the FRL treatment significantly decreased the relative expression of *TaCCR* gene. The relative expression of all genes was higher in SN23 than in SN16 during all stages of stem development, and the lowest relative expression of all genes was achieved at maturity.

### Correlation Analysis of Lodging Resistance With Stem Morphological Characteristics and Lignin Content

**Figure 8:**
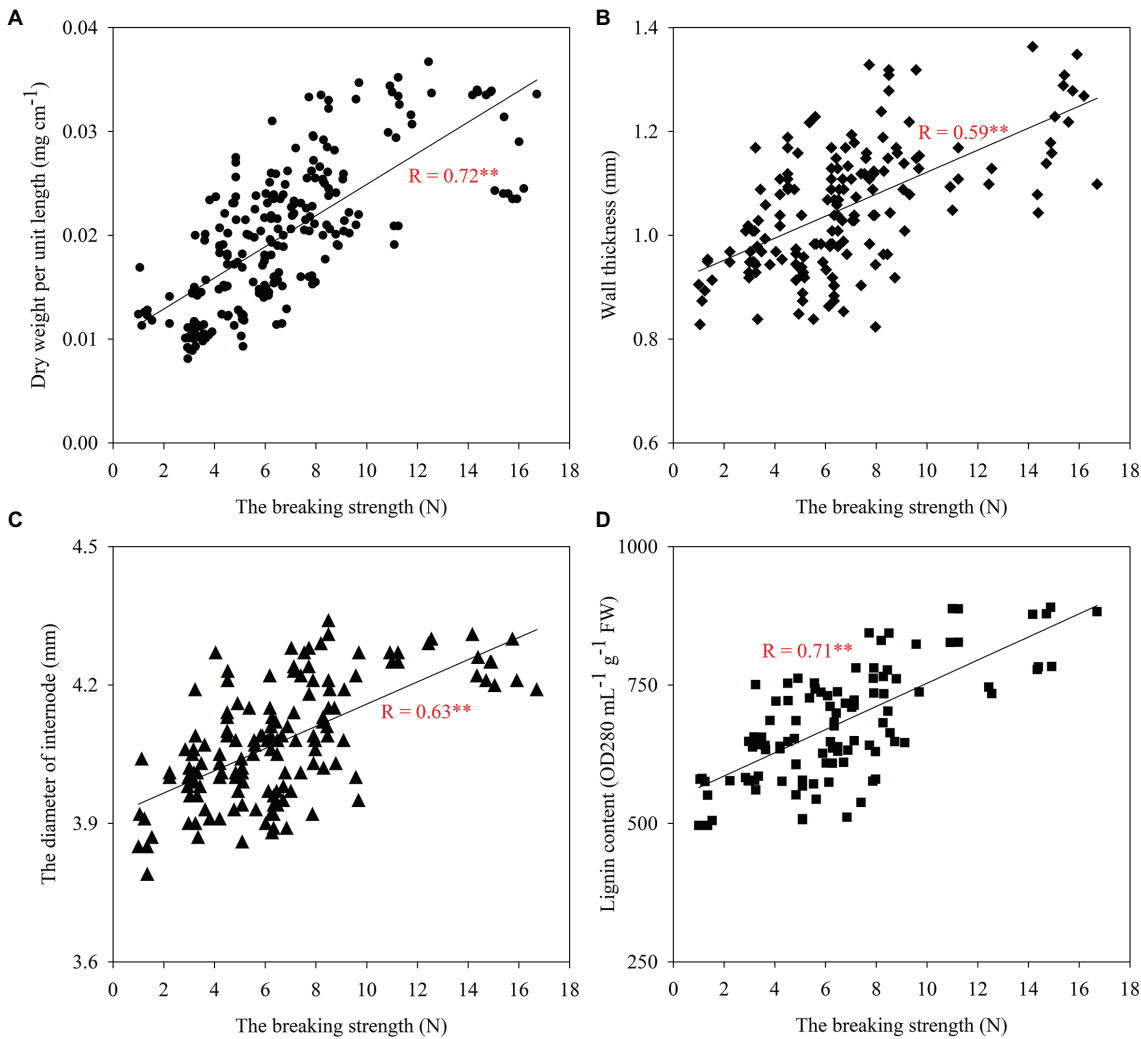
Correlation analysis between breaking strength and the dry weight per unit length **(A)**, the wall thickness **(B)**, the diameter of internode **(C)**, and the lignin content **(D)** of the second basal internode. Correlation coefficients (r) are calculated. Asterisks (*) represent significance at the 0.05 probability level (**A**: *n*=234, **B**: *n*=168, **C**: *n*=144, and **D**: *n*=108).

Stem morphological characteristics such as filling, diameter, and wall thickness of the second internode at the base of the stem were highly significantly and positively correlated with folding resistance (Figure 8). The correlation analysis also showed that the lignin content of the second internode at the base of the stem was highly significantly and positively correlated with folding resistance, and the proportion and distribution of lignin subunits affected the breaking strength of the stem, with the proportion of H subunits significantly and negatively correlated with the breaking strength of the stem, and the proportion of S+G subunits significantly and positively correlated with the breaking strength of the stem (Table 5). The results indicated that increasing the second internode filling, stem thickness, and wall thickness at the base of the wheat stem was beneficial to enhance its fracture resistance and increasing the lignin content and S+G subunit proportion at the base of the stem was beneficial to enhance the resistance of wheat to lodging.

**Table 5:** The relationship between the breaking strength and the lignin subunit percentage. H, G, and S represent p-hydroxyphenyl lignin, guajacyl lignin, and syringyl lignin, respectively.

	S (%)	G (%)	H (%)	S+G (%)	G+H (%)	S+H (%)	S/G
Breaking strength at anthesis stage	0.36^ns^	−0.06^ns^	−0.77^*^	0.77^*^	−0.36^ns^	0.06^ns^	0.19^ns^
Breaking strength at milk stage	0.68^ns^	−0.52^ns^	−0.77^*^	0.77^*^	−0.68^ns^	0.52^ns^	0.64^ns^
Breaking strength at dough stage	0.78^*^	−0.75^ns^	−0.80^*^	0.80^*^	−0.78^*^	0.75^ns^	0.77^*^

* *Represent significance at the 0.05 probability level*.

ns *Represent not significant at the 0.05 probability level*.

## Discussion

### Stem Morphological Characteristics

Plants sense changes in the light environment through photopigments, the intracellular distribution of which is influenced by light intensity and light quality. It is involved in various photomorphogenesis by participating in the R/FR reaction and influencing the growth process of plants. It regulates various photomorphogenic responses to optimize plant growth and morphogenesis by participating in the R/FR response (Smith, 2000; Rockwell et al., 2006; Fraser et al., 2021).

**Figure 9:**
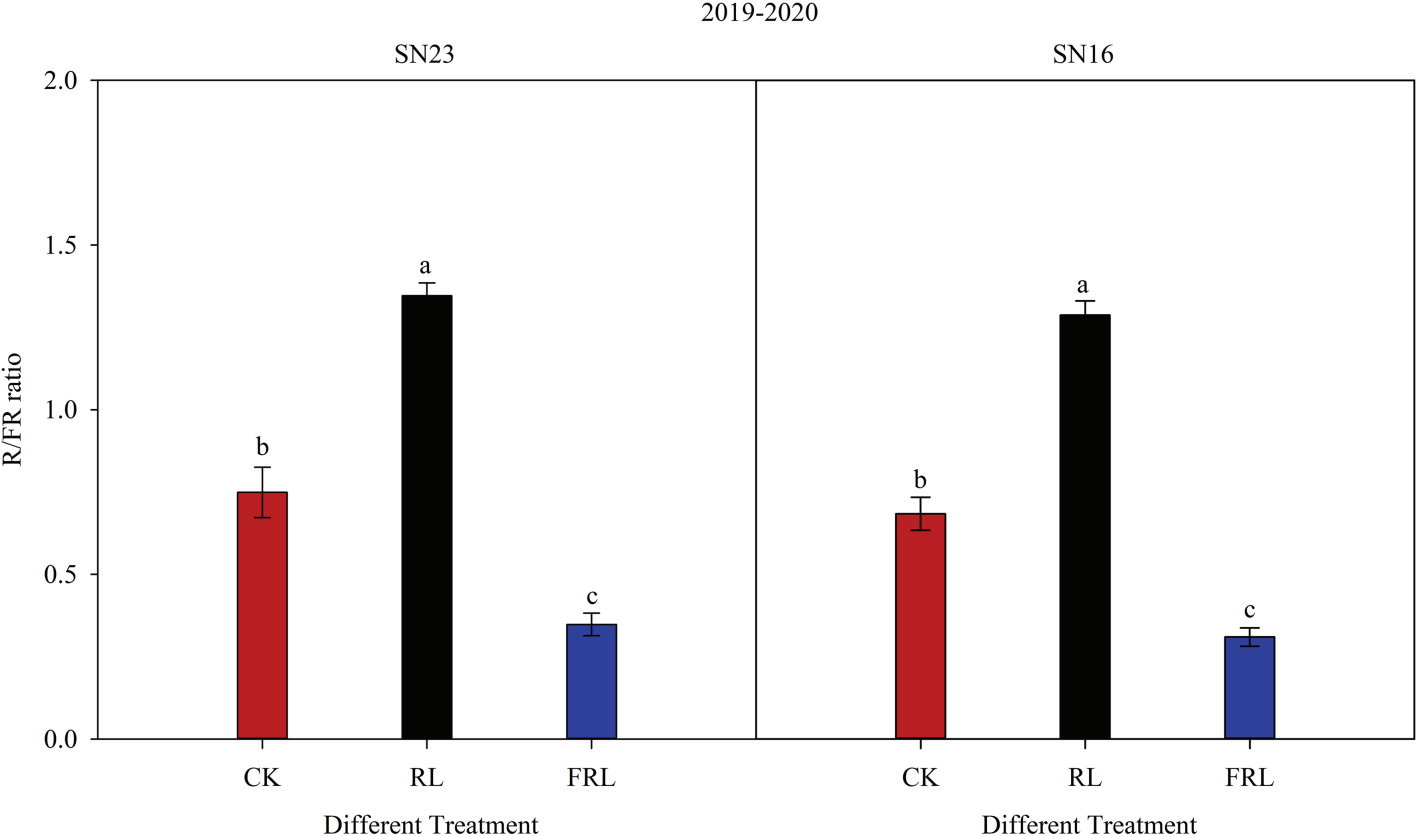
Red: far-red ratio (R/FR) at the base of canopy under different treatments. SN16 and SN23 represent the lodging-sensitive cultivar Shangnong16 and the lodging-resistant cultivar Shangnong23. CK represents the normal light, RL represents the red light, and FRL represents far-red light. Vertical bars above mean values indicate SDs (*n*=5). Means show significant differences between treatments according to the LSD (*p*<0.05).

The breaking strength of the internode at the base of the stem is closely related to the light environment, and this study showed that high R/FR conditions (Figure 9) significantly increased the diameter and wall thickness of the second internode at the base of the wheat stem (Figure 2) and increased stem fullness (Table 4), thereby significantly enhancing stem breaking resistance and improving the lodging resistance. This is due to the existence of two types of reactions in the photomorphogenesis process, namely the R/FR reaction and the blue light/near-ultraviolet light reaction. Among them, the proportion of R/FR has the greatest effect on plant photomorphogenesis (Smith, 1982; Park and Runkle, 2017). Among the many phytochromes, Phy A is the only FRL receptor in plants, and at low R/FR proportions, Phy A regulates the inhibition of shade avoidance responses leading to stem elongation, whereas, at high R/FR proportions, the inhibition for shade avoidance responses is mainly regulated by Phy B (Shlumukov et al., 2001; Zhou and Li, 2019). These findings confirmed that the reasons for the different expressions of *Phy A* and *Phy B* genes under different R/FR conditions (Figure 7). After the light environment changed, the distribution of phytochromes is altered to enter the nucleus to bind to some transcriptional regulators (Franklin and Quail, 2010; Wang and Zhang, 2017). In *Arabidopsis*, under low R/FR conditions, Phy B converts to Pr type, it transfers from the nucleus to the cytoplasm, thus enhancing the stability of PIF 4 growth regulators, at the same time, the cycling of Phy A between Pr and Pfr types compensates to some extent for the release of PIF 4 and prevents its degradation, which promotes the synthesis of GA (Tamotsu et al., 2005; Lorrain et al., 2007). Similar findings have been made recently in wheat (Pham et al., 2018; Sibbett, 2018). At the same time, in the condition of high R/FR, phytochrome inhibits the biosynthesis of GAs, and Phy A cooperates with Phy B to regulate the inactivation of GAs (Franklin, 2008). Stem elongation is one of the functions of GAs. The decrease of GAs concentration enhances the stability of DELLA protein, promotes the degradation of PIF, and finally inhibits stem elongation (Fumiaki et al., 2012; Balcerowicz, 2020). Analysis of the results with our studies confirmed that high R/FR conditions significantly reduced GA content (Figure 3).

Several reports show that increasing planting density results in lower R/FR at the base of the wheat stem, elongate growth at the base internodes, significantly reducing lodging resistance of wheat (Syros et al., 2005; Han et al., 2015), which is consistent with the results of Table 3. However, these findings were contrary to previous studies, which have suggested that the height of cucumber (*Cucumis sativus Linn*.), tomato (*Lycopersicon esculentum Miller*.), and ginger (Zingiber officinale Roscoe) plants were significantly increased under supplemental RL conditions, increasing the risk of lodging (Zhang et al., 2008; Cui et al., 2009). The difference in the results of this experimental study may be related to the differences between species.

### Lignin Metabolism

Gibberellin regulates lignin biosynthesis by affecting lignin monomer polymerization (Biemelt et al., 2004). Light regulates gibberellin biosynthesis and metabolism (Liu et al., 2021). RL inhibits the elongation of *Arabidopsis* stems by controlling the synthesis of GAs (Tamotsu et al., 2005; Liu et al., 2021). The effect of GAs on xylem lignin is less, but significantly reduces the lignin content of phloem. One study shows that stem lignin content is significantly reduced by spraying exogenous GA3 onto wheat (Peng et al., 2014). GA1, GA3, GA4, and GA7 exist commonly in plants as the primary bioactive forms of GAs (Hedden and Thomas, 2012; Tavares et al., 2018). GAs have different activities in different tissues/organs, e.g., embryo in germinating seeds of rice, young leaves in developing seedlings, the base of internode elongation (Silverstone and Sun, 2000). GA1 is the active GAs in soybean seedlings, GA4 is the active GAs in *Arabidopsis* hypocotyl, and GA3 plays a major role in seed germination (Kaneko et al., 2003; Falcioni et al., 2018). The GA7 content in wheat stems was remarkably high compared to GA4 in Figure 3, so we guessed that GA7 might be the active GAs in wheat stems. Some studies find that the GAs content and lignin content of dwarf plants are lower than those of wild-type plants, and the GAs content is positively correlated with the lignin content of stems (Biemelt et al., 2004; Okuno et al., 2014). Overexpression *gibberellin 2-oxidase (GA2oxs)* in switchgrass develop plants with low level of lignin (Wuddineh et al., 2015), whereas the lignin content of stems decreased when exogenous GA3 sprays on wheat (Peng et al., 2014). That could be that low levels of GAs increased lignin content and high levels of GAs inhibited lignin synthesis. In addition, our study also found that wheat varieties with different lodging resistance were consistent in their response to R/FR, but the GAs and lignin contents varied greatly. Interestingly, the lignin and GAs contents of the lodging-sensitive wheat under high R/FR conditions were maintained at the same level as those of the lodging-resistant wheat under CK. It indicates that lignin and GA contents are closely related to genotypes and regulated by environmental factors.

The analysis of the breaking strength of stems at different periods in wheat revealed that the breaking strength of stems decreased with the growth and development process (Table 3), while total lignin increased with the growth and development process (Figure 4). Surprisingly, that was contrary to a significant positive correlation between breaking strength and lignin content (Figure 8D). This result may be explained by the fact that stem breaking strength is not only correlated with lignin content, but also with other traits of the stem. Wheat straw is composed of 40% cellulose, 35% hemicellulose, and 25% lignin (Ruiz et al., 2013). In wheat, cellulose is an important factor in regulating the mechanical strength of the stem, which has implications in crop lodging (Kaur et al., 2017). Hemicellulosic arabinose reduces the ability of rice to lodging resistance by reducing cellulose crystallinity (Li et al., 2015). The low lignin content but higher breaking strength of stems in wheat during early growth and development may be due to the high pectin and water content inside the stem. Findings have shown that downregulation of switchgrass pectin biosynthesis gene *GAUT4* leads to reduced ferulic acid synthesis, lower cross-linking of lignin hydrate, and lower rigidity and fracture resistance of the stem, and these nonstructural substances are also important factors in determining the magnitude of the stem breaking strength (Handakumbura and Hazen, 2012; Li et al., 2019). Another important finding is that differences in the structure and proportion of lignin subunits determine their contribution to wheat stem resistance performance (Zheng et al., 2017a). The analysis of lignin subunits revealed that the proportion of H subunits also increased with fertility (Figure 6), and the proportion of H subunits was significantly and negatively correlated with the breaking strength of the stem (Table 5), which could also explain this phenomenon.

Phy A and Phy B promote the synthesis of lignin (Hernando et al., 2021). This study showed that high R/FR conditions significantly increased *TaPhy B* gene expression and significantly decreased *TaPhy A* gene expression (Figure 7). The activated state of photosensitized pigments activates the expression of related genes through G-protein, calmodulin, and cGMP pathways, which induce oxidative lignin deposition by regulating the flow of photosynthetic carbon sources to the lignin synthesis pathway (Anderson et al., 2015). The R/FR proportion at the base of the wheat stem may be a key factor in altering lignin synthesis and ultimately lignin composition, and a high R/FR proportion significantly increases lignin accumulation in wheat (Liu et al., 1996; Luo et al., 2019). Analysis of stem lignin subunit data revealed that high R/FR proportions significantly increased lignin subunit content and significantly reduced H subunit content and proportions (Figures 5, 6), with most of the reduced H subunit present as S and G subunits (Figure 5), and that R/FR significantly affected stem breaking strength (Table 3).

It has been suggested that the S/G proportion at the basal second internode of wheat stems was significantly and positively correlated with the stem breaking strength (Zheng et al., 2017a). However, some studies have shown that there were multiple possibilities for the relationship between the content and proportion of lignin subunits and the breaking strength of the stem (Luo et al., 2019). This study found that the content of G+S+H lignin subunits, the proportion of G+S lignin subunits to H subunits were the main factors affecting the breaking strength of wheat stems, and the stem breaking strength was significantly correlated with the S/G proportion only at the dough stage (Table 5).

### Gene Expression

Light affects lignin biosynthetic capacity at the transcriptional level, and the R/FR proportion ultimately affects lignin synthesis, subunit proportion, and distribution (Rogers et al., 2005; Mah et al., 2018), thus affecting stem lodging resistance performance. The current study found that a high R/FR proportion significantly increased the expression of lignin synthesis-related enzyme genes (Figure 7). The increase in lignin content was achieved by increasing the activity and gene expression of lignin biosynthetic enzymes in the lignin synthesis process. Genes involved in the early steps of the lignin biosynthesis pathway affect lignin content, while genes involved in the final steps alter lignin composition (Li et al., 2008; Yu et al., 2020). It has been shown that *PAL*, *CAD*, *HCT*, *COMT*, and *4CL* are significantly and positively correlated with lignin content in wheat (Boudet et al., 2003; Bi et al., 2010). Under shade conditions, the R/FR proportion decreased, thereby decreasing the expression of *CAD*, *4CL*, and *PAL* genes. The expression of lignin synthesis-related enzyme genes is significantly reduced in soybean and *Arabidopsis*, under low R/FR proportion (Rogers and Campbell, 2004; Hussain et al., 2020). Under lower R/FR conditions, *C3H*, *C4H*, *CCR*, *CCoAOMT*, and *POD* expression are downregulated in soybean, leading to a decrease in the content of lignin synthesis precursors such as caffeic acid, erucic acid, and ferulic acid, and alter lignin subunit content and proportion (Ren, 2017; Liu et al., 2018b).

In addition to the above genes, this study showed that the expression of *CSE* and *F5H* genes in the lignin synthesis pathway increased with the increase of R/FR proportion. *CSE* is a key enzyme for carbon flow partitioning in the lignin synthesis pathway, and F5H is a key enzyme for the synthesis of lignin S subunits and their free radicals. It has an important effect on lignin biosynthesis and molecular structure polymerization (Day et al., 2009; Stewart et al., 2009). Another important finding was that the expression of each gene reached its lowest level at the late stage of wheat growth and development, and the difference between treatments decreased due to the programmed cell death stage of wheat, where the stem lost its biological activity and lignin synthesis stopped, but high R/FR conditions could delay this decreasing trend (Figure 7). The most interesting finding was that most of the gene expression peaks occurred in the early stage of stem development and significantly decreased in the late stage of development, but lignin content continued to increase. A possible explanation for this might be that higher levels of gene expression occur in the early stages of stem development when the rate of lignin accumulation is high, and that lignin accumulates later in growth and development due to the stability of lignin that is difficult to break down, thus allowing lignification to be maintained.

**Figure 10:**
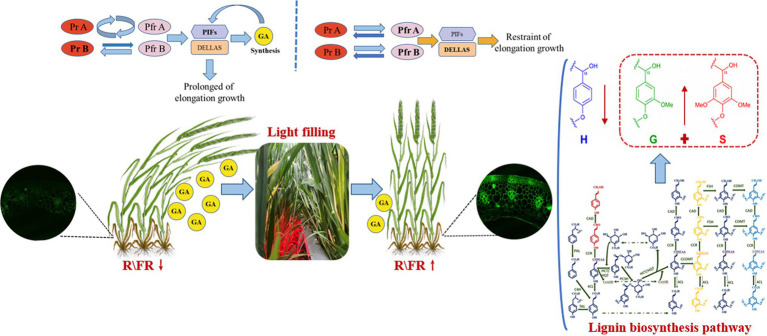
Hypothetical mechanism of high R/FR promotion of lignin biosynthesis.

We can have a bold guess that the light environment at the base of dense populations becomes poor (R/FR↓). Low R/FR condition makes phytochrome inactive (Franklin, 2008). Inactivated phytochrome leads to an increase in GAs content and a decrease in lignin content, resulting in slender stems and deteriorating stem quality (Tamotsu et al., 2005). After RL irradiation (R/FR↑), phytochrome transforms into the activation state (Pfr). The activation state (Pfr), which inhibits the shade avoidance response, leads to a decrease in GA content and an increase in the expression of keys enzyme genes for lignin synthesis, contributing to improved stem quality (Fumiaki et al., 2012; Balcerowicz, 2020). The hypothetical mechanism by which high R/FR promotes lignin biosynthesis is shown in Figure 10. Future genomic, transcriptomic, and proteomic analyses should reveal more details and deepen our understanding of the role of R/FR in crop growth and development.

## Conclusion

Wheat stems were sensitive to R/FR, and high R/FR favored lignin accumulation in wheat stems. High R\FR condition decreased the GA content of wheat stems, H subunit content, and H subunit proportion; increased total lignin content, S+G content, and S+G proportion. High R\FR led to higher stems fullness, increased stem thickness and wall thickness, higher breaking resistance, better stem quality, and reduced risk of lodging. This study aimed to provide a theoretical basis for regulating the light quality conditions at the base of wheat stems to enhance the resistance to lodging.

## Data Availability Statement

The original contributions presented in the study are included in the article/Supplementary Material, further inquiries can be directed to the corresponding author.

## Author Contributions

YL and Z-LW designed and planned the research. C-HL, S-FS, and MJ conducted the experiments. C-HL and Y-LL analyzed the data and wrote the manuscript. All authors contributed to the article and approved the submitted version.

## Funding

This study was supported by the Natural Science Foundation of Shandong Province (grant no.: ZR2020QC106) and the National Key Research and Development Program of China (grant nos.: 2017YFD0301001 and 2016YFD0300403).

## Conflict of Interest

The authors declare that the research was conducted in the absence of any commercial or financial relationships that could be construed as a potential conflict of interest.

## Publisher’s Note

All claims expressed in this article are solely those of the authors and do not necessarily represent those of their affiliated organizations, or those of the publisher, the editors and the reviewers. Any product that may be evaluated in this article, or claim that may be made by its manufacturer, is not guaranteed or endorsed by the publisher.

## Footnotes

^1^ http://www.ncbi.nlm.nih.gov/nuccore/

^2^ http://plants.ensembl.org

^3^ http://wheat-urgi.versailles.inra.fr/
