# Supplementary material for: Response of Lignin Metabolism to Light Quality in Wheat Population
Source: Front Plant Sci. 2021 Sep 13;12:729647. doi: 10.3389/fpls.2021.729647 (PMC8473876; doi:10.3389/fpls.2021.729647)
Supplement: Supplementary file 1 [file Table_1.DOCX]

TABLE S1 | The efficiency of primers and the copies of target genes.

E, PCR efficiency; R^2^ , regression coefficient.

| **Gene ID** | **The copies of target gene** | **E (%)** | **R^2^** |
| --- | --- | --- | --- |
| *β-ACTIN*  (TraesCS1A02G274400) | 2 | 90.1% | 0.994 |
| *phytochrome A (PHY A1)*  (TraesCS4A02G262900) | 2 | 100.8% | 0.995 |
| *phytochrome B (PHY B3)*  (TraesCS4A02G122500) | 2 | 95.3% | 0.978 |
| *phenylalanine ammonialyase (PAL4)*  (TraesCS6A02G222800) | 2 | 91.6% | 0.995 |
| *cinnamate 4-hydroxylase (C4H2)*  (TraesCS3B02G375100) | 2 | 95.3% | 0.975 |
| *p-hydroxycinnamoyl-CoA shikimate (HCT2)*  (TraesCS2B02G374600) | 2 | 104.2% | 0.971 |
| *p-coumarate 3-hydroxylase (C3H1)*  (TraesCS3D02G336900) | 2 | 116.5% | 0.98 |
| *cinnamoyl-CoA reductase (CCR2)*  (TraesCS5D02G232400) | 2 | 90.5% | 0.994 |
| *cinnamyl alcohol dehydrogenase (CAD4)*  (TraesCS6D02G162800) | 2 | 105.3% | 0.986 |
| 1. *coumarate:CoA ligase (4CL2)*   (TraesCS7D02G483400) | 1 | 101.8% | 0.96 |
| *ferulate 5-hydroxylase (F5H2)*  (TraesCS2B02G518400) | 2 | 93.7% | 0.984 |
| *caffeic acid O-methyltransferase (COMT1)*  (TraesCS3B02G612000) | 2 | 106.4% | 0.984 |
| *caffeoyl shikimate esterase (CSE)*  (TraesCS2B02G229300) | 2 | 96.5% | 0.998 |
